# Pathogenicity assessment and whole-genome sequencing of *Salmonella abortus equi* strain XJ2032 isolated from Xinjiang, China

**DOI:** 10.3389/fvets.2025.1698040

**Published:** 2025-10-27

**Authors:** Han Fu, Tongyang Li, Yuyan Wang, Yang Yang, Yabin Lu, Jianlong Li, Jianhua Liu, Ling Kuang, Zhanhai Mai, Qingyong Guo

**Affiliations:** ^1^College of Veterinary Medicine, Xinjiang Agricultural University, Urumqi, China; ^2^Xinjiang Key Laboratory of New Drug Research and Development for Herbivores, Urumqi, China

**Keywords:** *Salmonella abortus equi*, virulence, antimicrobial resistance, genome-wide analysis, pathogenicity analysis

## Abstract

**Introduction:**

Equine abortus salmonellosis, caused by *Salmonella abortus equi* (*S. abortus equi*), is a contagious disease primarily characterized by abortion in pregnant equine animals. Due to its high pathogenicity and increasing incidence, this disease has attracted significant scientific attention. While the causes of abortion in mares are multifactorial and may involve numerous pathogenic factors, the specific impact of *S. abortus equi* on the vaginal microecological environment and its pivotal role as the primary causative agent of abortion remain poorly understood.

**Results:**

Further analysis led to the successful isolation and identification of *S. abortus equi* from vaginal samples of aborted mares. A highly pathogenic isolate, designated as XJ2032, was selected for further analysis. To gain a more profound understanding of the functional genomic composition and genetic traits of this strain, whole-genome sequencing was conducted, and sophisticated bioinformatics techniques were employed to predict and annotate its gene sequences. Furthermore, animal model experiments, and PCR-based molecular biological detection methods were utilized to assess the virulence and drug resistance genes of the isolated strain XJ2032, further confirming its pathogenic potential.

**Conclusion:**

Whole-genome sequencing analysis confirmed that strain XJ2032 is indeed *S. abortus equi*. Although its genome structure is largely conserved, some rearrangements and inversions were identified. The strain harbors multiple virulence genes and drug resistance genes, including horizontally transferable genes and mobile genetic elements. These findings suggest that genomic islands and bacteriophages play a vital role in the pathogenicity and genetic diversity of *S. abortus equi*.

## 1 Introduction

Salmonellosis is a notable bacterial disease that causes substantial economic losses in various livestock industries, including pig farming, poultry, pigeon breeding, as well as the equine sectors (1–3). *Salmonella abortus equi*, also known as equi paratyphoid, primarily affects equidae, with abortion in pregnant mares being its hallmark feature. The disease is caused by several *Salmonella* species, notably *S. abortus equi*, *Salmonella Typhimurium* (*S. typhimurium*), *Salmonella Dublin* (*S. Dublin*), and—worth noting the potential redundancy—*Salmonella Enteritidis* (*S. enteritidis*) (4, 5). Among these, *S. abortus equi* stands as the primary etiological agent. Prior documentation has also delineated cases of donkey abortions and diarrhea in donkey foals attributed to *S. typhimurium*, either alone or in combination with *S. abortus equi* (6). Conversely, infections in horses caused by *S. Dublin* and *S. enteritidis* are rare and scarcely reported. Molecular biological techniques have significantly advanced epidemiological research, particularly in tracking the spread of infections (7). Furthermore, next-generation sequencing methods, such as whole genome sequencing (WGS) and comparative genomic analysis, have recently become powerful tools for studying the emergence and loss of specific virulence factors and antibiotic resistance genes.

The pathogenicity of *Salmonella* arises from a complex interplay of numerous virulence-associated factors, including virulence islands, plasmid-encoded determinants, structural components (such as pili and flagella), and enterotoxins. Enhancing diagnostic and preventive strategies is therefore a priority in the equine industry. Advances in molecular biology have significantly propelled epidemiological research and innovation (7). In particular, WGS technology has emerged as a powerful tool to investigate specific changes in virulence and antibiotic resistance genes. However, the limited availability of publicly accessible whole genome sequence data for *S. abortus equi* strains hampers a comprehensive understanding of their evolution and adaptability within equine populations.

In this study, WGS confirmed the isolated strain as *S. abortus equi*. Collinearity analysis revealed that while the genome structure of this strain was largely conserved, rearrangements and inversions were observed compared to other *Salmonella* species. The genome contained 683 virulence genes and 311 antibiotic resistance genes, including one horizontally transferable gene. Additionally, 15 mobile genetic elements, such as insertion sequences and transposons, were identified. Genomic islands (GIs) and phage-transmitted virulence factors were found to play a pivotal role in shaping the pathogenicity and genetic diversity of *S. abortus equi* strains.

## 2 Materials and methods

### 2.1 Samples and strains

In order to ensure experimental accuracy, the vulvas of all mares were thoroughly cleaned and disinfected. Subsequently, sterile vaginal swabs were used to collect vaginal secretion samples from both groups. These samples were separately stored in sterile, enzyme-free 5mL cryopreservation tubes and broth medium. The cryopreservation tubes were immediately frozen in liquid nitrogen and transported on dry ice to the laboratory for comprehensive analysis of the diversity of vaginal microbiota diversity. Samples preserved in broth medium were used for bacteriological examination, specifically for the isolation and purification of potential pathogens.

### 2.2 Isolation and identification of Salmonella abortus in horses

#### 2.2.1 Isolation and culture of strains

The vaginal swabs and samples of aborted fetal tissue samples were aseptically collected and carefully processed. The samples were cut into small pieces and inoculated into a liquid medium for pre-enrichment, followed by incubation at 37 °C with constant agitation at 180 rpm for 12–16 h. Subsequently, an inoculating loop was used to transfer the bacterial solution onto the solid medium for streak plating. The plates were then inverted and incubated for an additional 16–20 h, allowing for the formation of distinct bacterial colonies. In accordance with the principles of colony morphology, a solitary colony that had been adequately isolated was selected for inoculation into a fresh liquid medium. This inoculation process was repeated on three occasions, with incubation at 37 °C and 180 rpm agitation for 12–16 h during each cycle, in order to ensure the purity of the bacterial culture. Ultimately, the purified bacterial culture was preserved in glycerol with a view to maintaining its viability and integrity for subsequent use. The purification and preservation process was repeated on three occasions in order to guarantee the integrity and viability of the isolated strain.

#### 2.2.2 Bacterial morphological observations

The Gram staining procedure was performed in strict accordance with the instructions outlined in the Gram staining solution’s manual. A single colony, meticulously isolated through three rounds of purification, was carefully selected and subsequently incubated overnight at 37 °C with gentle agitation at 180 rpm to ensure optimal conditions for staining. Following this, the prepared slide was carefully heated to fix the bacteria and then thoroughly dried. The slide was subsequently examined under a light microscope to observe and document the detailed morphology of the bacterial cells.

#### 2.2.3 Identification of *16S rRNA, FliC, invA* gene via sequence analysis

The *16S rRNA, FliC* gene and *invA* gene sequence of *S. abortus equi* was retrieved from GenBank, and two pairs of highly specific primers were designed utilizing the Primer Premier 5.0 software. The sequences of these primers are as follows:16S rRNA-F: 5′-AGAGTTTGATCCTGGCTCA-3′,16S rRNA-R; ACCTGTCACCCGATGTACC. FliC-F: 5′-CCAGACTCAGTTCAACGG-3′, and FliC-R: 5′-AAACCGCCATCAATAGTC-3′. invA-F:5′-GTCACCGTGGTCCAGTTT-3′, and invA-R: 5′-CTCTTTCCAGTACGCTTCG-3′. These primers were synthesized by Shanghai Sangong Bioengineering Technology Service Co. The PCR reaction mixture had a total volume of 12.5 μL, comprising 6.25 μL of Taq DNA polymerase, 1 μL of DNA template, 0.5 μL of each primer (upstream and downstream), with ddH2O added to achieve the final volume. The reaction conditions were as follows: pre-denaturation at 95 °C for 5 min, 36 cycles of denaturation at 95 °C for 30 s, annealing at 56 °C for 30 s, and extension at 72 °C for 90 s, and total extension at 72 °C for 10 min (PCR for *16 S rRNA* gene); 95 °C pre-denaturation for 3 min, 34 cycles of denaturation at 95 °C for 30 s, annealing at 52 °C for 30 s, and extension at 72 °C for 30 s, and total extension at 72 °C for 10 min (double PCR for *FliC* and *invA* genes). The PCR products were subsequently analyzed by gel electrophoresis to identify positive samples. Selected positive bands were excised from the gel, and the DNA was recovered. The purified PCR products were sent to Shanghai Sangong Bioengineering Technology Service Co., Ltd. for sequencing. Following sequencing, the nucleotide sequences of the isolated strains were compared to related sequences in GenBank using the DNA Star software. Finally, a phylogenetic tree was constructed using the Mega7.0 software to further elucidate the genetic relationships among the strains.

### 2.3 Pathogenicity of the strain

Forty-two mice were randomized into seven groups, ensuring equal distribution of males and females within each group. Prior to inoculation, cultures of the XJ2032 strain, grown to the logarithmic phase at 37 °C, were carefully collected and washed twice with sterile phosphate-buffered saline (PBS).

The mice in the first group were inoculated with 1.5 × 10^8^ CFU/mL, the second group with 1.5 × 10^7^ CFU/mL, the third group with 1.5 × 10^6^ CFU/mL, the fourth group with 1.5 × 10^5^ CFU/mL, and the fifth group with 1.5 × 10^4^ CFU/mL. As a control, the mice in the sixth group received an equivalent volume of sterile PBS via intraperitoneal injection. The animals were then closely monitored for the onset of clinical symptoms and mortality, and the lethal dose (LD_50_) was determined based on these observations.

To assess histopathological changes, the heart, liver, spleen, lungs, and kidneys were harvested, fixed in 4% paraformaldehyde for 48 h, and subsequently stained with hematoxylin and eosin (HE). Histological alterations in these organs were compared to those observed in the control tissues.

The LD_50_ was calculated using the following method:

Distance ratio: This was determined using the formula (Mortality above 50–50%)/(Mortality above 50%–Mortality below 50%).

lgLD_50_: The logarithm of the bacterial dilution corresponding to the dose at which mortality exceeded 50% was calculated. The product of the logarithm of the distance ratio and the dilution factor was then added to determine the LD_50_ value.

### 2.4 Detection of virulence genes

The bacterial genome was used as the template to amplify 12 widely recognized virulence genes(*hilA, spvC, sipA, sopE, pefA, sipC, ssrA, sopB, sefA, rck, stn, ssaR*) and 10 drug resistance genes (*aac(6)ly, qnrA, aaC3, qnrS, blaTEM, parC, tetA, tetB, sul I, sul II*)specific to *Salmonella*. The sequences of the gene-specific primers used for this process are detailed in Supplementary Tables S1 and S2. These primers were synthesized by Sangon Bioengineering (Shanghai, China) Co., ensuring high quality and specificity. For the PCR amplification reaction, a reaction mixture of 12.5 μL was prepared, comprising 6.25 μL of 2 × Easy Taq PCR SuperMix (+dye), which contains the essential enzymes and reaction buffers for PCR performance; 0.5 μL of each upstream and downstream primer, carefully selected to ensure specific amplification of the target genes; 1 μL of DNA template, containing the bacterial genome to be analyzed; and 4.25 μL of ddH2O, bringing the total volume to 12.5 μL to ensure accurate and reproducible results. After PCR amplification, the gel-purified PCR products were sent to Shanghai Biotech for sequencing. The exact nucleotide sequences of the amplified genes were determined with high precision. This step is crucial for further analysis and identification of the virulence and drug resistance genes present in the bacterial genome.

### 2.5 MLST typing

The WGS of *S. abortus equi* XJ2032 was analyzed using the MLST 2.0 software, which allowed the extraction of sequences for seven housekeeping genes: *hisD, hemD, thrA, sucA, aroC, purE*, and *dnaN*. Subsequently, multilocus sequence typing (MLST) was performed using the PubMLST database.

### 2.6 WGS, assembly, and data quality control analysis

The genome of *S. abortus equi* XJ2032 was sequenced utilizing a *de novo* bacterial genome sequencing approach that combined the strengths of second-generation Illumina HiSeq and third-generation PacBio RS II technologies. Sequencing libraries for both platforms were prepared and processed using NovaSeq 6,000 and PacBio Sequel IIe sequencers, respectively. The sequencing data underwent rigorous quality control procedures, which included the removal of adapter sequences, trimming of the 5′ ends to eliminate non-A, G, C, and T bases, and trimming of low-quality read ends (specifically those with a quality value below Q20). Additionally, reads containing more than 10% N bases were removed, as were small fragments shorter than 25 bp after adapter removal and quality trimming. These quality control steps ensured the generation of high-quality sequencing data. Subsequently, the reads were assembled into contigs, and their circularity was assessed. This process ultimately resulted in the construction of complete chromosome and plasmid genome. All sequencing, assembly, and quality control procedures were carried out by Shanghai Meiji Biomedical Technology Co., ensuring high precision and reliability.

### 2.7 Statistical analysis

Correlation network analysis was carried out utilizing R (version 3.4.1) and Cytoscape, with a correlation threshold set at 0.3. This analysis allowed for the visual representation of relationships between species, metabolic functions, samples, and sample classifications. Key patterns and insights were derived from these network maps. Genome circle maps were generated using CGView (Version 2) software, providing a comprehensive visualization of the genomic structure. For visualizing the COG, GO, KEGG, and CAZy data, Origin 2021 software was used to create detailed and informative maps.

## 3 Results and analyses

### 3.1 Morphological observation of the isolated strains

**Figure 1:**
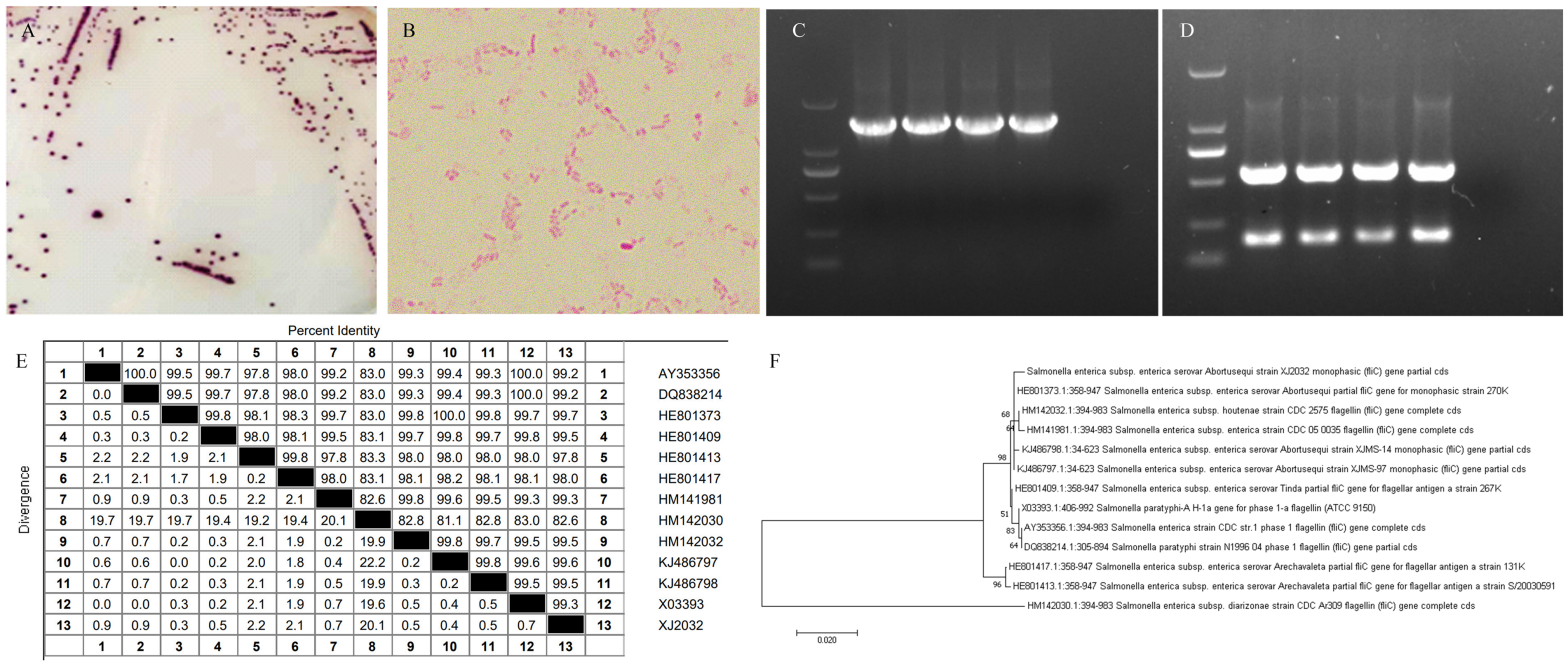
Isolation and identification of bacteria. **(A)** Colony morphology and morphological characteristics of the isolated strain on Salmonella-contained medium. **(B)** Gram staining results of the isolated strain. **(C)** PCR results of *16S rRNA* gene of the isolated strain. **(D)** Dual PCR results of *FliC* and *invA* genes of the isolate. **(E)** Nucleotide similarity comparison between the isolate and reference strain *FliC*. **(F)** Phylogenetic analysis of isolate *FliC* nucleotides.

In the present experiment, a bacterial pure culture method was utilized for the isolation and identification of bacteria from aborted fetal tissues and vaginal swabs of aborted mares. The isolated bacterial colonies were of medium size, round in shape, and exhibited distinct, raised purple colonies on *Salmonella* staining medium (Figure 1A). Following purification, the isolates were subjected to Gram staining. Microscopic examination revealed that the bacteria were Gram-negative, short bacilli with bluntly rounded ends and no visible spores (Figure 1B).

### 3.2 *16S rRNA, FliC, invA* gene sequence identification results

A distinct single band was visualized through 1% agarose gel electrophoresis of the PCR product targeting the *16S rRNA* gene of the isolate (Figure 1C). The dual PCR product of the *FliC* and *invA* genes from this isolate also showed two distinct bands on 1% agarose gel electrophoresis (Figure 1D). Subsequent sequencing of this PCR product and comparison with sequences of S. abortus equi in the NCBI database revealed a sequence identity of 99% or higher, confirming the identity of the isolate as *S. abortus equi*. Consequently, the strain was designated as XJ2032. To delve deeper into the genetic characteristics, the *FliC* gene sequences of two isolates were aligned with sequences deposited in GenBank utilizing DNASTAR and MEGA7 software. This alignment provided a comprehensive analysis of the homology between the *FliC* gene of various *S. abortus equi* isolates and those available in GenBank. As illustrated, the nucleotide sequence similarity of the *FliC* gene between the Salmonella equorum isolate from Yili, Xinjiang, and other *Salmonella* isolates ranged from 82.6 to 99.7% (Figure 1E). Notably, the sequence similarity between this isolate and the Irish isolate HE801373 reached a high of 99.7%, aligning with the findings of the similarity analysis presented (Figure 1F). Detailed information on the reference strains is provided in Supplementary Table S3.

### 3.3 Pathogenicity test results in mice and growth kinetics

**Figure 2:**
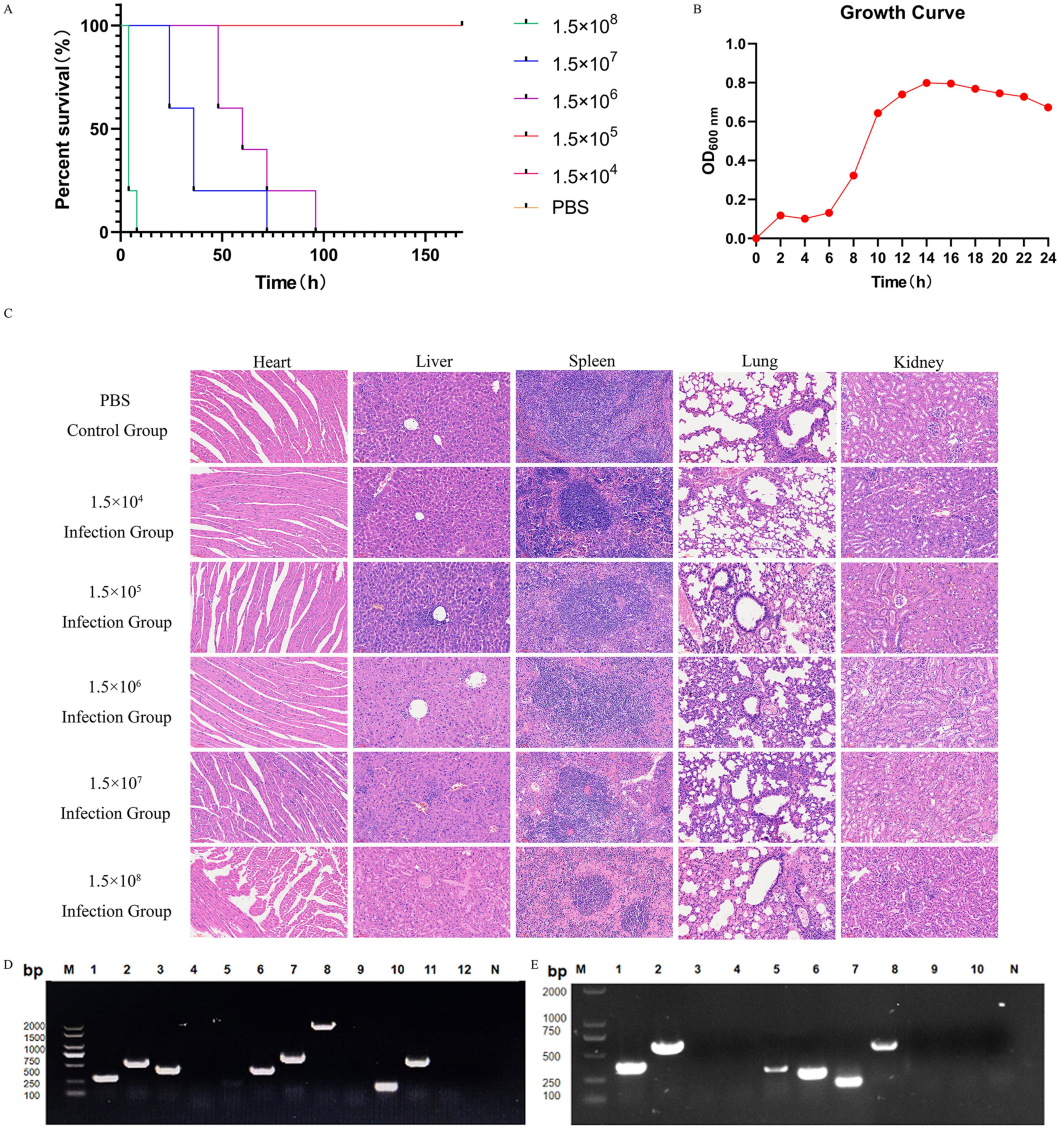
Pathogenicity and virulence genes of the isolate strains, and results of resistance gene testing. **(A)** Survival curves of the isolated strains causing death in mice. Differences in survival curves were evaluated by the log-rank test. **(B)** Growth curve of strain XJ2032 inoculated in MM broth and cultured at 37 °C. The optical density at 600 nm (OD_600_) of the bacterial suspension was determined every 2 h. Data are presented as the mean and standard deviation from independent experiments. **(C)** Histopathological examination of different visceral tissues and organs collected from deceased mice following infection. HE staining of visceral tissues of mice in the PBS-treated group and under different concentration gradients is shown, from top to bottom, respectively (×40). **(D)** PCR amplification results for 12 virulence genes in the strain. Lane designations: M: Marker; 1: *hilA*; 2: *spvC*; 3: *sipA*; 4: *sopE*; 5: *pefA*; 6: *sipC*; 7: *ssrA*; 8: *sopB*; 9: *sefA*; 10: *rck*; 11: *stn*; 12: *ssaR*; N: Negative control. **(E)** PCR amplification results for 10 drug resistance genes in the strain. Lane designations: M: Marker; 1: *aac(6)ly*; 2: *qnrA*; 3: *aaC3*; 4: *qnrS*; 5: *blaTEM*; 6: *parC*; 7: *tetA*; 8: *tetB*; 9: *sul I*; 10: *sul II*; N: Negative control.

In the mouse infection model, animals in the group inoculated with 1.5 × 10^8^ CFU/mL of the isolate succumbed to the infection within 24 h. In contrast, only one mouse in the group administered 1.5 × 10^7^ CFU/mL died, while the remaining mice in all groups exhibited symptoms such as unkempt fur and lethargy within 1 week of infection. The control group, however, remained healthy throughout the experiment (Figure 2A). A single colony of *S. equi abortus* (XJ2032 strains) was injected into 5 mL of MM and cultured for 24 h with shaking at 37 °C. To assess growth, the OD _600_ was measured and recorded every 2 h (Figure 2B).

Subsequently, the visceral tissues of the infected mice were collected and preserved using the modified Reed–Muench method, allowing for the calculation of the LD_50_ of the isolate, which was determined to be 4.74 × 10^5^ CFU/mL. Histopathological examination was then conducted to further investigate the effects of the infection (Supplementary Table S4).

HE staining revealed significant pathological differences between the infected and control groups. Specifically, the heart tissues of the infected mice showed varying degrees of inflammatory cell infiltration. Similarly, the liver tissues of the infected mice exhibited inflammatory cell infiltration, along with cytoplasmic cell changes, including pale cytoplasm, scattered eosinophilic alterations, disordered arrangement of hepatic cords, narrowed hepatic sinusoids, and areas of coagulative necrosis in some hepatic tissue.

The spleen tissues of the infected mice also showed significant pathology, including obvious hemorrhage, macrophage hyperplasia, lymphocyte edema, disorganized and distorted medullary cords, and substantial inflammatory cell infiltration. Likewise, the lung tissues of the infected mice displayed varying degrees of alveolar wall thickening, accompanied by hemorrhage, progressive loss of normal lung structure, severe inflammatory cell infiltration with neutrophils predominating, alveolar wall hyalinization, and aggregation of red blood cells within the alveoli, leading to thrombus formation. In conclusion, the renal tissues of infected mice exhibited a range of pathological changes, including glomerular swelling, vascular endothelial cell hyperplasia, obstruction of capillary lumens, and significant inflammatory cell infiltration. The cytoplasm of renal cells appeared loose and lightly stained, with multiple vacuoles. Some renal capsule lumens contained leukocytes, red blood cells, and other exudates, further indicating significant renal damage (Figure 2C).

### 3.4 Detection results of virulence and resistance genes

A comprehensive PCR analysis was conducted on the isolated strain, XJ2032, to screen for 12 common *Salmonella* virulence genes and 10 drug resistance genes. The results revealed that strain XJ2032 harbored 8 virulence genes, corresponding to a detection rate of 66.7%. Additionally, 6 drug resistance genes were identified, yielding a detection rate of 60%. The detailed results of this analysis are illustrated (Figures 2D,E).

### 3.5 MLST typing results

Seven housekeeping genes from the isolated strain XJ2032 were sequenced, and the resultant sequences were submitted to the PubMLST database, a publicly accessible platform for molecular typing and microbial genome diversity. Based on the analysis of the sequence data in the database, the sequence type (ST) of strain XJ2032 was determined. The findings revealed that strain XJ2032 belonged to ST-251, a sequence type associated with *S. abortus equi*, as outlined in Table 1.

**Table 1:** MLST typing results.

hisD	hemD	thrA	sucA	aroC	purE	dnaN	ST
89	18	87	96	93	5	4	ST-251

### 3.6 WGS results

**Figure 3:**
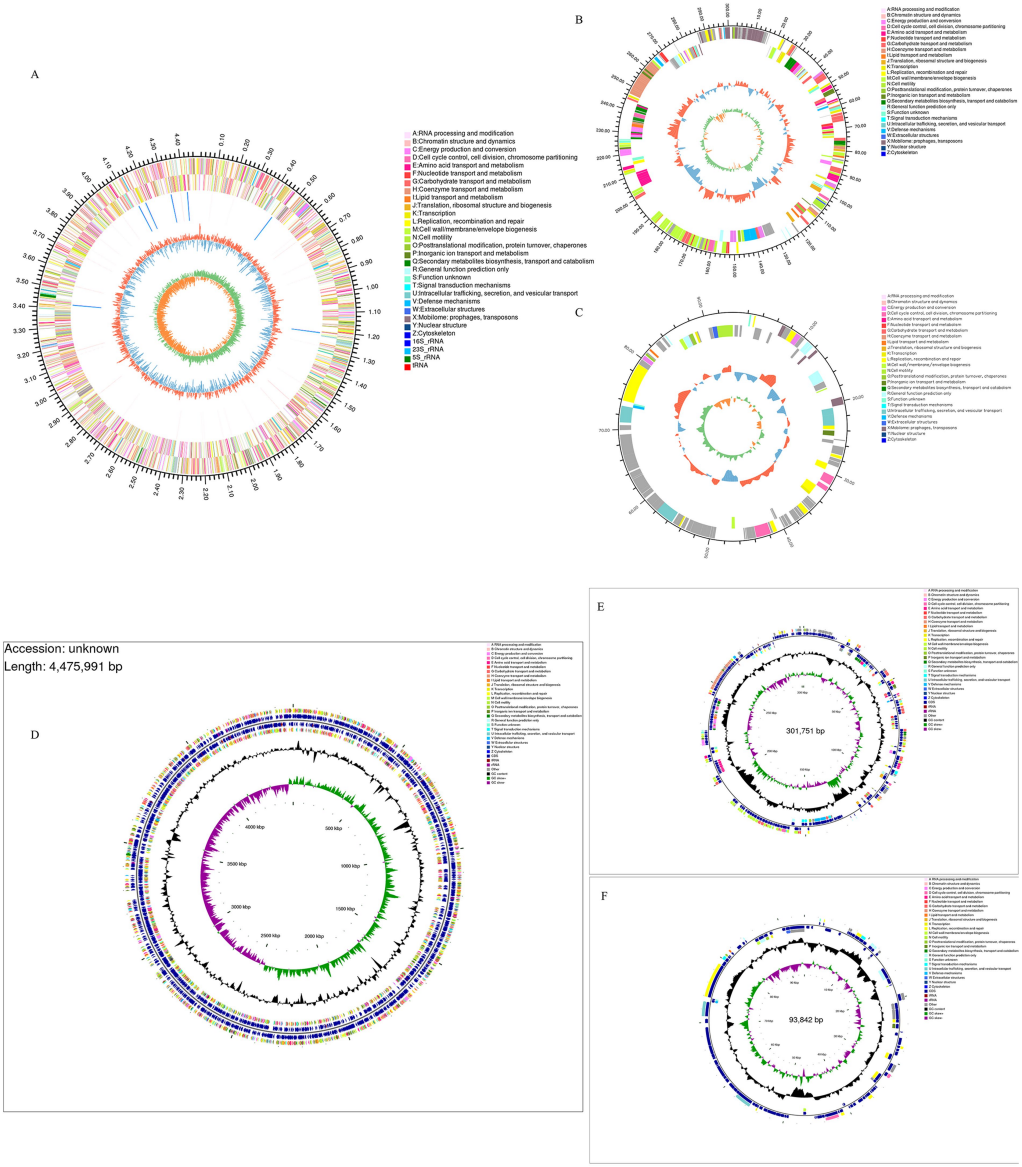
XJ2032 genome ring map, Genome CGView map. The outermost circle represents the genome size. The second and third circles display the coding sequences (CDS) on the positive and negative strands, respectively, with different colors indicating the functional classification of the CDSs based on COGs. The fourth circle shows the positions of rRNAs and tRNAs. The fifth circle represents the GC content, where the outward red portion indicates regions with GC content higher than the average for the entire genome, with higher peaks reflecting greater deviation from the average GC content. Conversely, the inward blue portion indicates regions with GC content lower than the genome’s average. The blue inward part has higher peaks corresponding to greater differences from the average GC content. The innermost circle represents the GC-Skew value, calculated as G − C/G + C. This value helps identify the leading and lagging strands of the genome, where the GC skew of the leading strand is >0 and that of the lagging strand is <0. The GC-Skew can also assist in determining the origin (cumulative offset minimum) and the termination (cumulative offset maximum) of replication.

The whole genome of strain XJ2032 was sequenced using a combination of second- and third-generation sequencing technologies, enabling the construction of a comprehensive genome circular map. The genome spans 4,871,584 base pairs (bp) in length and consists of one chromosome and two plasmids, both in circular form. The chromosome is 4,840,545 bp in length (Figure 3A), while the unnamed plasmid (Plasmid 1) is 301,751 bp (Figure 3B), and the plasmid pRSE04 is 93,842 bp (Figure 3C). Together, these genomic elements encode a total of 4,743 genes. The GC content of the genome is 52.15%, reflecting the proportion of guanine (G) and cytosine (C) nucleotides (Figures 3D–F). A total of 4,764 coding genes were predicted, with a combined length of 4,202,406 bp, and the average gene length is 899.1 bp, accounting for 86.26% of the total genome sequence. Notably, the genome contains 16 gene islands (gIs), 7 prophages, and 7 CRISPR (Clustered Regularly Interspaced Short Palindromic Repeats) loci, all of which contribute to genome plasticity, virulence, and defense mechanisms. Additionally, the genome encodes 75 tRNAs (transfer RNAs) and 22 annotated rRNAs (ribosomal RNAs), both critical for protein synthesis. Furthermore, 204 bacterial small RNAs (sRNAs) were identified, which play crucial roles in regulating various cellular processes, including outer membrane protein expression, iron homeostasis, community sense, and bacterial pathogenicity. These findings highlight the complexity and regulatory potential of the XJ2032 genome, genome island, pre-phage details are shown in Supplementary Figures S1 and S2.

### 3.7 Multi-strain covariate analysis

**Figure 4:**
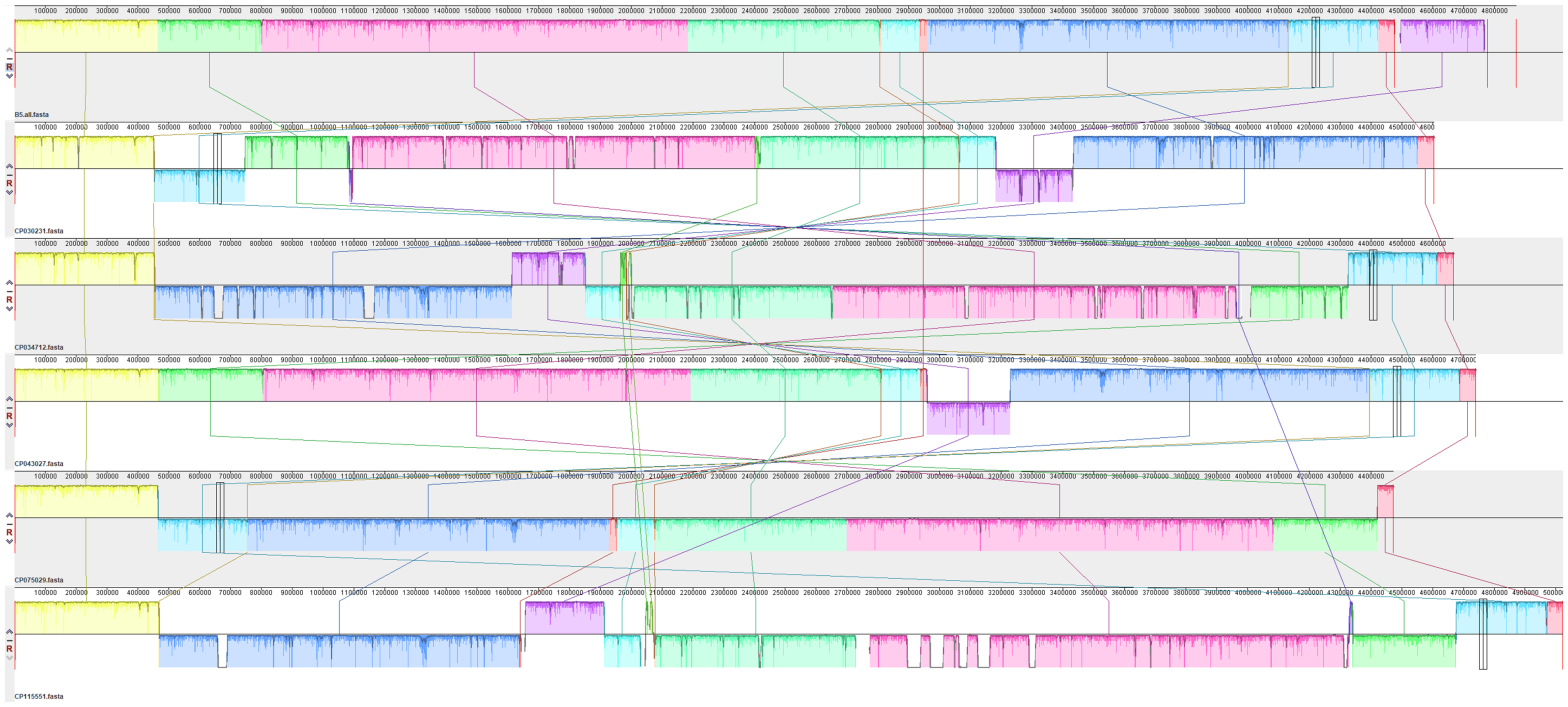
Collinearity analysis results of isolated strains and other strains. The collinearity analysis of bacterial genomes shows that the isolated strains exhibit rearrangements or inversions compared to other *Salmonella*.

The homogeneity of the genome was further analyzed utilizing Mauve to compare the chromosomal structures of the international reference strains of the isolates, focusing on their covariance. The resulting genome structure is depicted in Figure 4. When compared to the standard strain of *S. abortus equi*, a greater number of homologous genes were identified, suggesting a globally more homogeneous genome. However, in contrast to other *Salmonella* genomes, more complex variations were observed, indicating the presence of rearrangements and inversions among genes within each strain (Figure 4).

### 3.8 Results of genome sequence analysis

**Figure 5:**
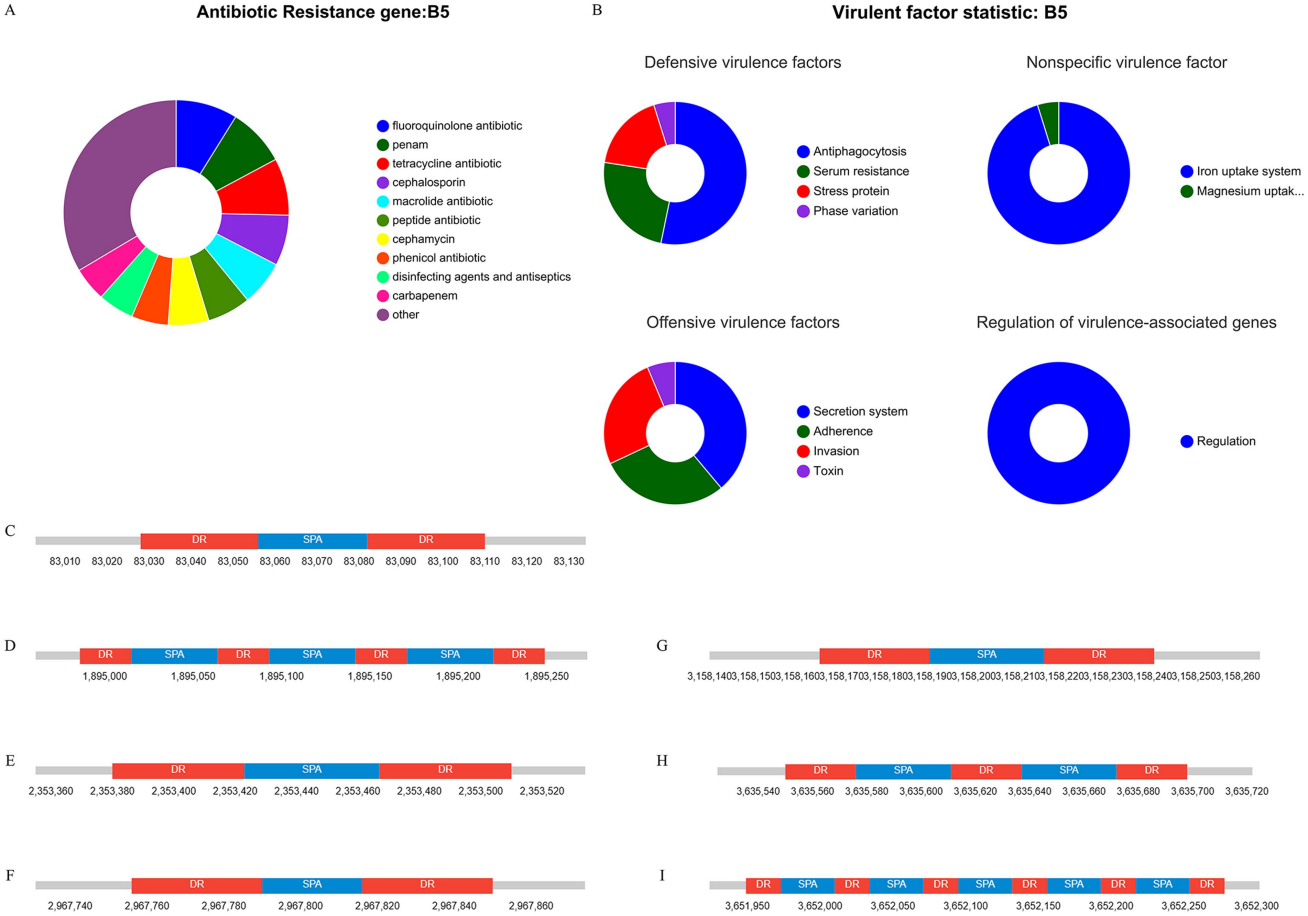
Results of genome sequence analysis. **(A)** Statistical table of resistance gene prediction. **(B)** Categorical statistical map of virulence gene prediction. **(C–I)** CRISPR-Cas1-7 prediction structure diagram.

The CARD database analysis revealed that strain XJ2032 contained a total of 311 resistance genes, with the majority related to the multidrug efflux system located on the chromosome (Figure 5A). Notably, one horizontally transferable aminoglycoside resistance gene, *aac(6′)-Iaa*, was identified on the chromosome, exhibiting 98.17% sequence similarity. This strain demonstrated the highest resistance to fluoroquinolones, followed by resistance to 38 other antibiotics, including carbapenems and tetracyclines, which was consistent with the results of the preliminary antimicrobial susceptibility testing. These findings suggest that strain XJ2032 is multidrug-resistant. Further confirmation of aminoglycoside resistance genes was obtained from the ResFinder prediction results. Additionally, bacterial mobile genetic elements (MGEs), such as insertion sequences (IS) and transposons (Tn), were analyzed for their role in the horizontal transfer of resistance genes. These MGEs facilitate the rapid spread of drug resistance between different bacterial species. Using ISEScan and Transposon PSI, we identified 15 MGEs in strain XJ2032. The multidrug-resistant phenotype of *S. abortus equi* XJ2032, with resistance to 38 antimicrobials, was primarily attributed to the action of the efflux pump system (Supplementary Table S5).

Comparative analysis with the VFDB database revealed that strain XJ2032 harbored 683 virulence genes. These genes mainly encode proteins involved in key functions such as adhesion, iron uptake, bacterial secretion systems, and cell membrane formation (Figure 5B).

The CRISPR/Cas system is a prokaryotic immune mechanism that defends against the invasion of exogenous genetic elements, such as phage viruses and plasmids. This system recognizes foreign DNA and suppresses the expression of exogenous genes. CRISPR (Clustered Regularly Interspersed Short Palindromic Repeats) comprises a vast array of short, conserved repeats interspersed with spacer regions. Adjacent to the CRISPR loci, Cas proteins-specifically double-stranded DNA nucleases are responsible for cleaving target DNA with the assistance of guide RNA. Together, the CRISPR repeats and Cas proteins form the CRISPR-Cas system (Figures 5C–I).

### 3.9 Functional annotation and prediction of genomes

**Figure 6:**
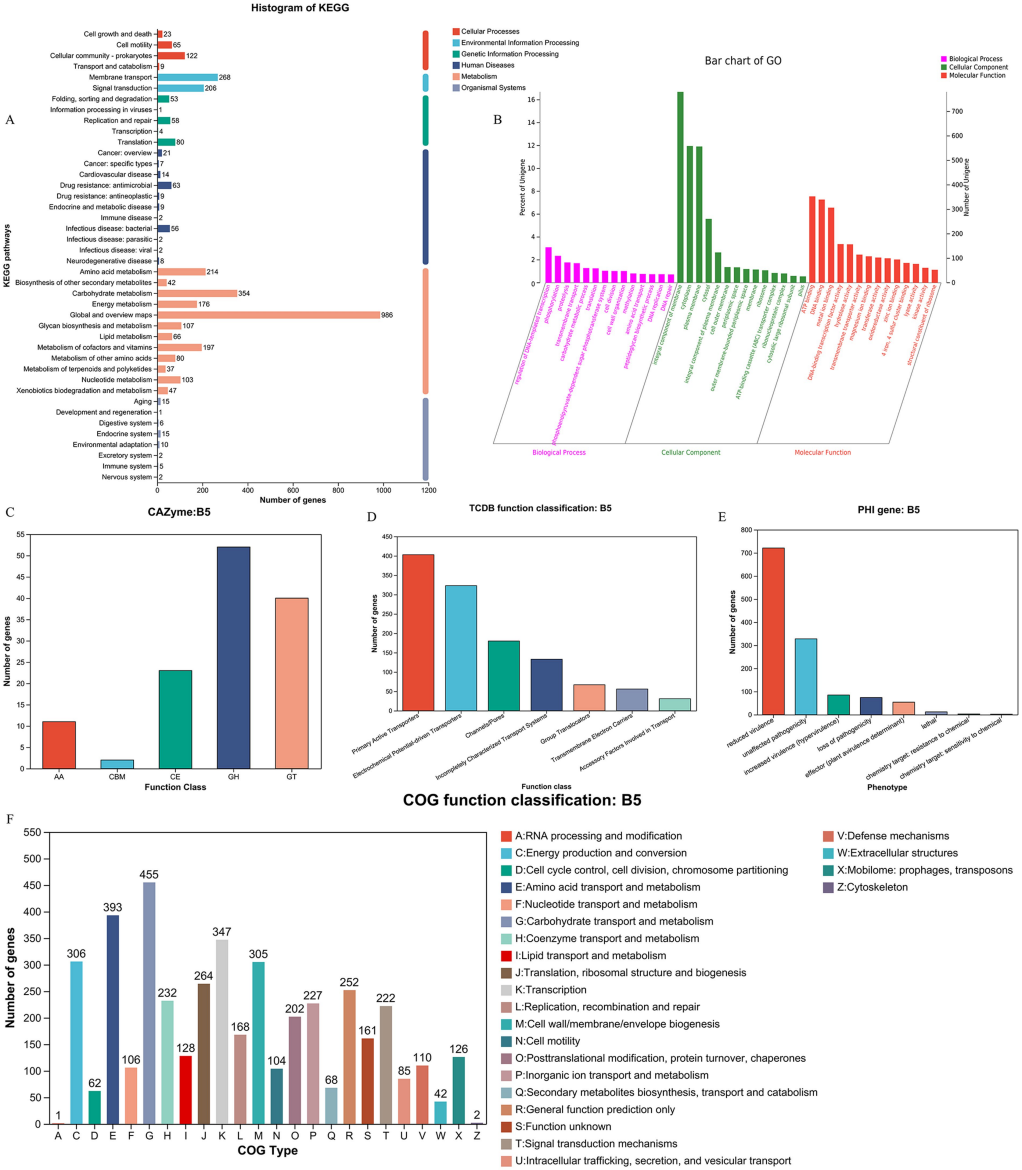
Functional annotation and prediction of the genome. **(A)** KEGG functional classification of the *S. abortus equi* XJ2032 genome. **(B)** Functional classification of the *S. abortus equi* XJ2032 genome GO. **(C)** Functional classification of the *S. abortus equi* XJ2032 genome CAZy. **(D)** Functional classification of the *S. abortus equi* XJ2032 genome TCDB. **(E)** Statistics on the number of genes with different PHI phenotypic mutation types in the *S. abortus equi* XJ2032 genome. **(F)** Functional classification of COG in the *S. abortus equi* XJ2032 genome.

Utilizing the KEGG database, we conducted a comprehensive analysis of the metabolic pathways and gene functions associated with strain XJ2032. A total of 3,547 genes were annotated, all encoding proteins involved in various KEGG pathways. Notably, the majority of these genes (2,409) are implicated in metabolic pathways, with significant enrichment in the global overview (986 genes), carbohydrate metabolism (354 genes), and amino acid metabolic pathways (214 genes). Additionally, 474 genes were found to be involved in the environmental information processing pathway, predominantly enriched in membrane transporter (268 genes) and signal transducer (206 genes) pathways. Furthermore, 352 genes were linked to genetic information processing. It is important to note that the original text contained a repetition regarding the number of genes in the genetic information processing pathway (474 genes), followed by a reference to 196 genes. For clarity, we have interpreted the latter number (196 genes) as a subset or further categorization within this broader pathway. Furthermore, 219 genes were associated with cellular processes, 193 with human diseases, and 56 with organic systems. Particularly within the human disease pathway, we identified metabolic pathways related to antimicrobial resistance (63 genes) and bacterial infectious diseases (56 genes), both of which are crucial for understanding the pathogenicity and drug resistance of the XJ2032 strain (Figure 6A).

Following annotation with the GO database, we identified a total of 3,332 genes in the genome of strain XJ2032 were annotated, representing 49.5% of the total gene count. These annotated genes were predominantly associated with metabolic processes, cellular processes, and localization. In the classification of cellular components, 2,015 genes were annotated, most of which are related to cells and cellular components. In terms of molecular function, 2,754 genes were annotated, with the majority linked to catalytic activity and binding. Furthermore, in the biological process classification, 2,225 genes were annotated. The results of the cellular component annotation revealed that *S. abortus equi* XJ2032 had a strong ability to form biofilms, which serve as a protective barrier against environmental stressors. The genes involved in localization are primarily associated with bacterial adhesion, a key factor that facilitates the colonization of the host cell surfaces and enhances the strain’s invasive potential (Figure 6B).

We conducted a comprehensive analysis of enzyme families involved in the synthesis, breakdown, and modification of complex carbohydrates using the CAZy database. This analysis identified a total of 128 carbohydrate enzyme family genes within the strain. Among these, 52 genes were annotated as glycoside hydrolases, accounting for 40.6% of the total annotations. Additionally, 40 glycosyltransferases were identified, representing 31.2% of the annotated genes. A further 23 sugar esterase genes were found, comprising 17.9% of the total annotations. Lastly, 2 carbohydrate binding modules were also annotated. These findings indicate that strain XJ2032 is equipped with a robust set of enzymes, particularly glycoside hydrolases and glycosyltransferases, which play pivotal roles in the degradation, modification, and synthesis of complex carbohydrates (Figure 6C).

Utilizing the TCDB database, we categorized the membrane transporter proteins of strain XJ2032. The annotation revealed a total of 1,193 genes categorized at the first level. Notably, active transporters and electrochemically potentially driven transporters were the most prevalent, accounting for 33.8 and 27.1% of the total annotated genes, respectively. These findings suggest that the primary transport mechanism in this bacterium is based on active transport, with ion channel transport serving as a supplementary role (Figure 6D).

We conducted a comprehensive toxicity and pathogenicity analysis using Diamond software, comparing the amino acid sequences of *S. abortus equi* XJ2032 with those in the Pathogen-Host Interaction (PHI) database. By integrating the genes of the target species with their corresponding functional annotations, we obtained detailed insights into the phenotypic mutations associated with pathogenicity. The analysis revealed that the most common mutation types were those associated with virulence reduction, followed closely by pathogenicity-neutral mutations (Figure 6E).

We conducted a detailed analysis of the annotated protein sequences of *S. abortus equi* XJ2032 using the COG database to predict the primary functions of the corresponding proteins. The annotation results, presented in Figure 6A, revealed a total of 4,368 COG functional genes. Among these, 347 genes were associated with transcription (K), accounting for 7.9% of the total annotations. Additionally, 393 genes were associated with amino acid transport and metabolism (E), and 455 genes were involved in carbohydrate transport and metabolism (G), representing 8.9 and 10.4% of the total annotations, respectively. Other functional annotations were also well-represented, including translation, ribosome structure, and biosynthesis (J, 6.4%), cell wall/membrane/envelope biogenesis (M, 6.9%), signal transduction mechanisms (T, 5.1%), cofactor enzymes transport and metabolism (H, 5.3%), and inorganic ion transport and metabolism (P, 5.2%) (Figure 6F).

## 4 Discussion

Equine abortion diseases have been demonstrated to cause significant economic losses to the equine industry. While environmental factors, feeding management, and bacterial factors all contribute to abortion in horses, bacterial causes are the primary factor (8). Due to the intracellular parasitism of certain pathogens, such as *Salmonella*, latent infections can occur, meaning that outwardly healthy mares may harbor and shed the bacteria into the environment (9). Monitoring *Salmonella* is particularly important in equine herds, but compared to other pathogens, Salmonella has a longer incubation time and slower colony growth rate, making it easier to contaminate the environment and more difficult to isolate the bacteria for diagnosis. Therefore, in this experiment, we isolated the bacteria from the aborted fetal tissue samples. The results of these isolations were further validated with sequencing data, providing a more comprehensive understanding of the potential causative agents responsible for abortion in mares. WGS was used to obtain the complete genomic sequences of the isolates, which were accurately typed using the SeqSero 1.2 database.

*Salmonella* SPI acquires its virulence factors by horizontal gene transfer (HGT) (10). Whole genome analysis has become an essential approach for understanding the multiple virulence factors of *Salmonella*. In this study, the WGS of strain XJ2032 revealed 683 virulence genes. The mouse pathogenicity test confirmed that strain XJ2032 exhibited strong virulence. To further investigate, PCR detection was performed for common virulence and resistance genes, including plasmid-carried virulence genes, bacterial hair genes, enterotoxin genes and so on, based on previous studies. The final detection rate for virulence genes was 66.7%, and for resistance genes, it was 60%, which is relatively lower than that reported in similar studies (11). This discrepancy may be due to the low-copy nature of most virulence and resistance genes, resulting in low or absent gene expression. The genes related to the type III secretion system on the virulence islands SPI-1 and SPI-2 of *S. abortus equi* were particularly well-preserved, encompassing genes involved in adhesion, invasion, flagellin, ferric iron carrier ions, secretory effector proteins, and toxins (12). In this study, whole genome sequencing (WGS) of *S. abortus equi* XJ2032 was conducted, resulting in the identification of a genome length of 4,871,584 bp, comprising one chromosome and two plasmids. The chromosome size was 4,840,545 bp, while the plasmid unnamed1 and pRSE04 were 301,751 and 93,842 bp, respectively. A total of 4,743 genes were annotated, with a GC content of 52.15%. For genomic comparison, we used the reference *S. abortus equi* standard strain CP075029, the donkey-derived *S. abortus equi* isolate AD19 (CP043027), and two other *Salmonella* species. Our analysis revealed that the three *S. abortus equi* strains exhibited a high degree of covariance with each other, but significant genomic rearrangements and inversions were observed when compared to the other two reference genomes.

Viruses that infect bacteria are known as phages (13). They are functionally related to various aspects of bacterial lifestyle, adaptation, virulence, evolution and pathogenicity (14, 15). In the genome of *S. abortus equi* XJ2032, several phage-related elements play a role in its virulence. One such element is the presence of *gogB*, a virulence factor associated with the type III secretion system (T3SS) (16). *Salmonella* contains two distinct sets of T3SS: SPI-1, which encodes T3SS-1 (17), and SPI-2, which encodes T3SS-2 (18). T3SS-1 is crucial for the initiation of *Salmonella* invasion by facilitating contact between the bacterium and the cell membrane of the non-phagocytic host, through which effector proteins are injected (19–21). These effector proteins include structural changes in the host membrane, forming pleated structures that promote bacterial internalization and the formation of Salmonella-containing vesicles (SCVs). As a result, SPI-1 plays a key role in the invasion of epithelial cells (21, 22). Upon contact with host cells, SPI-1 secretes a number of effector proteins that are involved in processes such as host cytoskeletal rearrangement, immune cell recruitment, cellular metabolism, fluid secretion, and modulation of the host inflammatory response (23, 24). T3SS-2 is activated in SCV (18) and translocates effector proteins across the SCV membrane into the host cytoplasm (25). These proteins manipulate SCV transport and maturation, enhancing intracellular *Salmonella* survival and proliferation (26).

Some studies have highlighted the critical role of virulence genes in the infection and pathogenicity of *Salmonella*. For example, the virulence island SPI gene facilitates *Salmonella* invasion of the host and its intracellular proliferation, while the virulence plasmid spv gene assists *Salmonella* survival within macrophages and aids in the colonization of deeper tissues (27). Based on the report of Sun et al. (28), the detection of the SPI gene (*sopB*) and the spv gene (*spvC*) in the present study suggests that strain XJ2032 has a strong potential for infecting equine animals, suggesting that the isolate may possess a high risk to evade host defenses and exhibit strong pathogenicity. *S. abortus equi* XJ2032 can alter the actin cytoskeleton of intestinal epithelial cells via the T3SS-encoded *Salmonella* invasive proteins (*Sips*) and *Salmonella* outer proteins (*Sops*) on SPI-1, leading to membrane folding and bacterial internalization (29–31). Among them, SopE is capable of inducing host nitrate production, thereby promoting the growth of Salmonella in host cells (32). The type III secretion system plays a crucial role in invading host cells and secreting effector proteins, which contribute to the formation of Salmonella-specific SCVs (33). Additionally, the spvB gene, detected in this study, plays an important role in the later stages following bacterial uptake. Macrophages undergo apoptosis-like cell death due to actin cytoskeletal instability in host cells, which correlates with increased activation of caspase-3 (34). This process has been shown to require the *SpvB* gene (35). More recently, CRISPR-Cas technology has emerged as a powerful tool for studying bacterial pathogenicity. It allows targeted editing of bacterial genomes, enabling the investigation of specific virulence-associated genes (36). Collectively, the virulence factors of *S. abortus equi* XJ2032 promote its colonization and spread within the host, particularly in the equine vaginal tissues.

*Salmonella abortus equi* XJ2032 is a multidrug-resistant strain that carries a total of 311 resistance genes, most of which are chromosomally encoded and related to the multidrug efflux system. Notably, the strain contains a horizontally transferable aminoglycoside resistance gene, aac(6′)-Iaa, on its chromosome, exhibiting a 98.17% similarity to known sequences. The strongest resistance was observed to fluoroquinolones, followed by resistance to 38 other antibiotics, including carbapenems and tetracyclines. These findings align with the results of a drug sensitivity test conducted by Zhanhai et al. (37). Many microbial genomes contain MFS transporters, which typically function as single-component pumps that can export multidrug-resistant efflux pumps, such as AcrAB, MdtK, AcrEF, EmrB, and MdfA, through the bacterial membrane. These efflux pumps, when overexpressed, are known to export quinolones (38, 39). One of these efflux pumps has recently been identified as a key factor in the primary fluoroquinolone resistance mechanism (40). Of these, tetracyclines are broad-spectrum antibiotics, and resistance to this class typically occurs through two main mechanisms: the active exocytosis of antimicrobials by bacterial efflux pumps (e.g., *ramA*), and the alteration of the ribosome-binding site to inhibit protein synthesis (e.g., *tetB*) (41). In the present study, the sulI gene, detected in *S. abortus equi* XJ2032, shows structural similarities to sulII and sulIII. This gene interferes with the synthesis of dihydrofolate by competing with p-aminobenzoic acid for dihydrofolate synthase, thereby inhibiting bacterial growth. A study by Sun (28) et al. found that nine Salmonella equorum abortus strains, derived from donkeys, were 100% resistant to streptomycin and 33% resistance to amoxicillin, while three strains showed intermediate resistance to amoxicillin. The *qnr* genes are commonly associated with resistance to other classes of antibiotics, such as *β*-lactams and aminoglycosides (42). In the present study, the *qnr* gene was primarily associated with aminoglycosides, suggesting that Enterobacteriaceae strains carrying qnr resistance may pose a significant threat. Furthermore, quinolones are antibiotics that target bacterial type II topoisomerases, specifically DNA gyrase and DNA topoisomerases IV (43). These enzymes, encoded by the *gyrA*, *gyrB*, *parC*, and *parE* genes, are involved in regulating DNA supercoiling. Inhibition of these enzymes by quinolones disrupts DNA replication, leading to bacterial cell death (44–46) Changhwan et al. (47) demonstrated that antibiotic treatment of host cells infected with *S. typhimurium* resulted in continued secretion of typhoid toxin by antibiotic-resistant *S. typhimurium*.

Bacteria within biofilms are known to exhibit enhanced antimicrobial resistance, and infections caused by biofilm-producing bacteria are often resistant to antimicrobial chemotherapy (48). In addition, *Salmonella* biofilms contain several types of extracellular polymeric substances (EPS), including polysaccharides, proteins, nucleic acids, and matrices, all of which play crucial roles in biofilm formation and antimicrobial resistance (40). In the present study, the investigation focused on the genes contained within the genome GI03, which were found to encompass biofilm formation regulators. This finding suggests that bacteria capable of colonizing the host through biofilm formation may possess a competitive advantage in establishing persistent infections (49).

The results of this study showed that the *S. abortus equi* XJ2032 exhibited the highest resistance to fluoroquinolones, followed by resistance to 38 other antibiotics, including carbapenems and tetracyclines. Consequently, the resistance spectrum of the strains was relatively narrow, with notable resistance to amoxicillin (penicillin) and streptomycin (aminoglycosides). This resistance profile differs significantly from that of *Salmonella* strains of equine origin reported by Leon et al. (50) and Weese et al. (51). These differences may be attributed to variations in serotypes, the types of antibiotics used, and the regional mechanisms of resistance development in the prevalent strains.

## 5 Conclusion

In this study, a Gram-negative bacillus was isolated from a horse farm in the Ili region of Xinjiang, where abortion was occurring. After isolation, identification, and MLST typing, the bacterium was identified as *S. abortus equi*, belonging to the ST-251 type, and was named XJ2032. WGS showed that this strain was highly virulent, exhibited multi-drug resistance, and carried multiple virulence genes. In pathogenicity studies conducted on Kunming mice, the LD_50_ of the XJ2032 strain was found to be 4.74 × 10^5^ CFU.

The WGS of strain XJ2032 indicated strong potential infectivity in equids, suggesting that this isolate may have a high capacity to evade host defenses and possess significant pathogenicity. Resistance gene analysis showed that strain XJ2032 exhibited the highest resistance to fluoroquinolones, followed by resistance to 38 other antibiotics, including carbapenems and tetracyclines, with the potential for aminoglycoside resistance gene transfer. The genome of strain XJ2032 exhibits enhanced carbohydrate metabolism enzymes supporting nutrient acquisition and biofilm formation, active transport systems ensuring efficient material exchange, a toxicity-related mutation network explaining pathogenicity phenotypes, and an enrichment of antimicrobial resistance genes indicating potential clinical challenges. These findings not only deepen our understanding of *Salmonella* equistaphoci’s metabolic adaptation and pathogenic mechanisms, but also provide systematic genomic evidence for antibiotic resistance control and the development of novel therapeutic targets. The observed differences from previous reports may be attributed to variations in serotypes, the types of antibiotics used, and the mechanisms of resistance development in endemic strains within the region. WGS of strain XJ2032 provides valuable data that can aid in further exploring its pathogenic mechanisms and contribute to the development of vaccines.

## Data Availability

The datasets presented in this study can be found in online repositories. The names of the repository/repositories and accession number(s) can be found in the article/Supplementary material. Genome-wide data of the isolated strains in this study have been uploaded to NCBI, Acession number PRJNA1201343.
