# Supplementary material for: Pathogenicity assessment and whole-genome sequencing of Salmonella abortus equi strain XJ2032 isolated from Xinjiang, China
Source: Front Vet Sci. 2025 Oct 27;12:1698040. doi: 10.3389/fvets.2025.1698040 (PMC12599123; doi:10.3389/fvets.2025.1698040)
Supplement: Supplementary file 1 [file Supplementary_file_1.pdf]

frontiers

# Pathogenicity Assessment and Whole-Genome Sequencing of *Salmonella Abortus equi* Strain XJ2032 Isolated from Xinjiang, China

## *Supplementary Material*

## 1 Supplementary Materials and methods

### 1.1 Reagent

HB4092 *Salmonella* Chromogenic Medium was purchased from Qingdao Haibo Biotechnology Co., Ltd. DNA Marker 2000 (CW0623M), Taq DNA Polymerase (CW0690M), Bacterial Genome DNA Extraction Kit (CW0552S), and Agarose Gel DNA Recovery Kit (CW2302M) were all purchased from Kangwei Century Biotechnology Co., Ltd. All additional reagents used were of domestic analytical-grade purity.

### 1.2 Instruments

The experimental setup utilized the following equipment: a constant temperature oscillator (Shanghai Tiancheng Experimental Instrument Manufacturing Co., Ltd.), a DZKW-D-2 constant temperature water bath (Beijing Yongguangming Medical Instrument Co., Ltd.), an automatic autoclave sterilizer (Model: HVE-50), a centrifuge (Eppendorf AG 22331 Hamburg), an SW-CJ-2F double-sided vertical clean workbench (Shanghai Medical Equipment Factory Ltd.), a TProfessional PCR instrument (Biometra), a DYY-6C electrophoresis instrument (Beijing Liuyi Instrument Factory), and a BIO-RAD gel imaging system (Model: Universal Hood II).

### 1.3 Forecasts and Functional Notes

Gene prediction was conducted on the genome using Glimmer software (Version 3.02) to determine the distribution and structural organization of genomic genes. Subsequently, functional annotation of the predicted genes was carried out by integrating several databases through sequence comparison, encompassing COG, GO, KEGG, and Carbohydrate-Active Enzymes (CAZy). To ensure accurate and meaningful annotations, sequence alignments were conducted with a stringent E-value of ≤ 1e-10 across all databases.

## 2 Supplementary Figures and Tables

**Supplementary Table S1**

Primer information for PCR amplification of Salmonella virulence gene

| Primer name | Primer sequence (5´→3´) | amplification length |
|---|---|---|
| *hilA-F* | CGTGAAGGGATTATCGCAGT | 296 |
| *hilA-R* | GTCCGGGAATACATCTGAGC | |
| *spvC-F* | ACTCCTTGCACAACCAAATGCGGA | 571 |
| *spvC-R* | TGTCTCTGCATTTCGCCACCATCA | |
| *sipA-F* | CCATTCGACTAACAGCAGCA | 449 |
| *sipA-R* | CGGTCGTACCGGCTTTATTA | |
| *sopE-F* | CGAGTAAAGACCCCGCATAC | 362 |
| *sopE-R* | GAGTCGGCATAGCACACTCA | |
| *pefA-F* | ACGCTGCCAATGAAGTGA | 225 |
| *pefA-R* | CAGAAGCCCAGGTGATAGTG | |
| *sipC-F* | AGACAGCTTCGCAATCCGTT | 446 |
| *sipC-R* | ATTCATCCCTTCGCGCATCA | |
| *ssrA-F* | CTTACGATTACGCCATTTACGG | 706 |
| *ssrA-R* | ATTTGGTGGAGCTGGCGGGGACT | |
| *sopB-F* | CCTCAAGACTCAAGATG | 1987 |
| *sopB-R* | TACGCAGGAGTAAATCGGTG | |
| *sefA-F* | GCAGCGGTTACTATTGCAGC | 321 |

| | | |
|---|---|---|
| *sefA-R* | TGTGACAGGGACATTTAGCG | |
| *rck-F* | AACGGACGGAACACACAGAGTC | 189 |
| *rck-R* | TGTCCTGACGAAAGTGCATC | |
| *stn-F* | TTGTCTCGCTATCACTGGCAACC | 617 |
| *stn-R* | ATTCGTAACCCGCTCTCGTCC | |
| *ssaR-F* | GTTCGGATTTGCTTCGGG | 251 |
| *ssaR-R* | TCTCCAGTGACTAACCCTAACCAA | |

**Supplementary Table S2**

Primers for PCR amplification of Salmonella resistance genes

| Primer name | Primer sequence (5′→3′) | amplification length |
|---|---|---|
| *AAC(6')-Iy-F* | AACAAAACCGATCTGGAG | 260 |
| *AAC(6')-Iy-R* | CTTCAAATCCTAATGCCTG | |
| *qnrA-F* | TTGCCAGGCACAGATCTTGAC | 582 |
| *qnrA-R* | AAGAGGATTTCTCACGCCAGG | |
| *aaC3-F* | GTTACACCGGACCTTGGA | 674 |
| *aaC3-R* | ACGACATTCGTCAACTGCAA | |
| *qnrS-F* | CTGCAAGTTCATTGAACAGGGTG | 430 |
| *qnrS-R* | TCTAAACCGTCGAGTTCGGCG | |
| *blaTEM-1-F* | GTGCTGCCATAACCATGAGT | 282 |
| *blaTEM-1-R* | CTGCAACTTTATCCGCCTCC | |

| | | |
|---|---|---|
| *parC-F* | CTATGCGATGTCAGAGCTGG | 260 |
| *parC-R* | TAACAGCAGCTCGGCGTATT | |
| *tetA-F* | GCTACATCCTGCTTGCCTTC | 210 |
| *tetA-R* | CATAGATCGCCGTGAAGAGG | |
| *tetB-F* | CAGTGCTGTTGTTGTCATTAA | 571 |
| *tetB-R* | GCTTGGAATACTGAGTGTTAA | |
| *SulⅠ-F* | CGGAGTGAGCTACCTGAACA | 435 |
| *SulⅠ-R* | GACGCTCGCGTGGAGCTCCG | |
| *SulⅡ-F* | ATCAGAGGAAGATCATCTAAGG | 315 |
| *SulⅡ-R* | GATCCAGACAGCTCATCAATCC | |

**Supplementary Table S3**

Reference strain information for genetic evolution analysis

| Strain name | Genbank Login Number | source (of information etc) |
|---|---|---|
| Salmonella enterica (S. enterica) | HM141981 | United States of America |
| Salmonella enterica (S. enterica) | HE801417 | Irish |
| Salmonella arechavaleta (S. arechavaleta) | HE801409 | Irish |
| Salmonella enterica (S. enterica) | HM142032 | United States of America |
| Salmonella paratyphi (S. paratyphi) | DQ838214 | Australia |
| Salmonella enterica (S. enterica) | AY353356 | United States of America |
| Salmonella enterica (S. enterica) | HM142030 | United States of America |
| Salmonella enterica (S. enterica) | HE801373 | Irish |
| Salmonella paratyphi (S. paratyphi) | X03392 | United States of America |
| Salmonella enterica (S. enterica) | HE801413 | Irish |
| Salmonella enterica (S. enterica) | KJ486797 | China |
| Salmonella enterica (S. enterica) | KJ486798 | China |
| Salmonella enterica (S. enterica) | SAMN45933799 | China |

**Supplementary Table S4**

The lethal effect of different doses of Salmonella abortus equi on mice

Based on the Reed-Muench method, the $LD_{50}$ value of *Salmonella equi abortus* in mice was calculated to be 4.74 × 10^5 CFU/mL.

| Inoculation dose (CFU/mL) | 1.5 × 10^8 | 1.5 × 10^7 | 1.5 × 10^6 | 1.5 × 10^5 | 1.5 × 10^4 |
|---|---|---|---|---|---|
| Dilution of bacterial solution | 10^8 | 10^7 | 10^7 | 10^5 | 10^4 |
| Number of inoculated mice | 5 | 5 | 5 | 5 | 5 |
| Number of surviving rats | 0 | 0 | 0 | 5 | 5 |
| Number of dead rats | 5 | 5 | 5 | 0 | 0 |
| Cumulative total deaths | 15 | 10 | 5 | 0 | 0 |
| Cumulative total survival | 0 | 0 | 0 | 5 | 10 |
| death ratio | 15/15 | 10/10 | 5/5 | 0/5 | 0/10 |
| mortality rate | 100 | 100 | 100 | 0 | 0 |

**Supplementary Table S5**

Results of analysis of mobile genetic elements

| **MOBILEELEMENT NAME** | family-run Family | Kind Species | locus (of a gene) Position |
|---|---|---|---|
| **ISPlge3** | IS150 | Insertion sequence | 199187 - 200003 |
| **-** | IS200 | Insertion sequence | 779941 to 780658 |
| **-** | IS200 | Insertion sequence | 995748 to 996465 |
| **-** | IS200 | Insertion sequence | 1800475 to 1801192 |
| **ISSen1** | IS3 | Insertion sequence | 1999309 - 2000620 |
| **-** | IS200 | Insertion sequence | 2183031 - 2183749 |
| **ISSen1** | IS3 | Insertion sequence | 3882365 - 3883676 |
| **ISSen1** | IS3 | Insertion sequence | 3920544 to 3921855 |
| **chain00001** | LINE | Transposon | 1224815～1225228 |
| **chain00002** | mariner | Transposon | 1715643 to 1716092 |
| **chain00003** | gypsy | Transposon | 3167324～3167812 |
| **chain00004** | mariner_ant1 | Transposon | 274496 to 274753 |
| **chain00005** | ISC1316 | Transposon | 11852-12172 |
| **chain00006** | mariner_ant1 | Transposon | 19538-20374 |
| **chain00007** | gypsy | Transposon | 22178 - 22780 |

## Supplementary Figures S1 Genomic Island Linear Mapping

Bacterial GIs are distinct genomic regions that enable horizontal gene transfer between bacterial species, playing crucial roles in bacterial survival and pathogenesis. A detailed analysis of these islands revealed a wide variety of functional genes: GI01 contains genes related to the type VI secretion system, including *impL*, which encodes the membrane subunit *TssM*, *impM*, encoding a protein related to the type VI secretion system-related structural domain, and *symE*, a gene associated with the type I toxin. GI02 encodes genes involved in biosynthesis regulation, including transcriptional regulators specific to activated chlorine, and glycosyltransferases, among others. GI03 harbors genes crucial for biofilm formation, such as biofilm-forming regulators, FAD-binding proteins, glutathione S-transferase family proteins, acyl-coenzyme A/acyl-ACP dehydrogenases, and more. GI04 includes genes for proteins such as nicotinic acid phosphoribosyltransferase, site-specific integrases, PD-(D/E) XK nuclease-like structural domain proteins, phagosomal proteins, and the SPI-2 type III secretion system effector *SseI*. GI05 encodes various products, including glycine-tRNA ligase subunit α and β, molybdenum pterin guanine dinucleotide-containing S/N-oxide reductase, glyoxylate/hydroxypyruvate reductase (*GhrB*), and the outer membrane elicitor protein *LpfC*. GI06 contains genes for the SPI-1 type III secretion system guanine nucleotide exchange factor *SopE2*, and a DMT family transporter protein. Chromosome-located virulence islands such as GI07, GI08, GI09, and GI10 encode proteins, including the Gram-negative bacterial porin *OmpS1*, nucleotidyltransferases involved in DNA repair and cytochrome c biogenesis, putative proteins, non-specific acid phosphatases, and NAD (P)-dependent ethanol dehydrogenase. Additionally, plasmid-located virulence islands such as GI15, GI16, and other plasmid-located virulence islands encode a diverse range of proteins, including HD family hydrolases, DNA-adenine methylases, phage-encoded nucleic acid endonucleases, and phage endopeptidases, which are involved in cytolysis or viral release from the host cell (Fig S1).

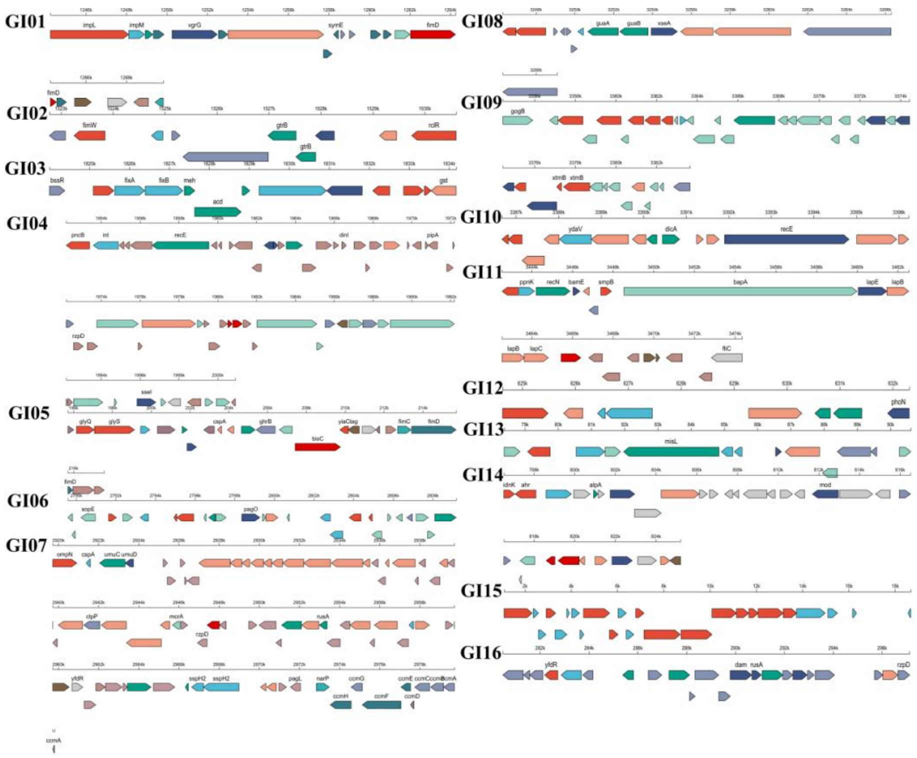

## Supplementary Figures S2 Pre-phage Lineage Tup

The integration of a phage genome into the bacterial genome results in the formation of a prophage, and bacteria that harbor such prophage genomes are referred to as lysogenic bacteria. These prophages frequently carry functional genes, such as antibiotic resistance genes and virulence genes, which enhance the bacteria's adaptability to its environment or contribute to its pathogenicity. Under certain physicochemical or biological stimuli, prophages can spontaneously excise from the host bacterial genome, entering the lytic cycle. During this cycle, mature phages are produced, leading to the lysis of the host bacterial cell. Among the various prophages identified, Ph01 contains genes encoding glycosyltransferase family 2 proteins, which are involved in the biosynthesis of cell wall components. Ph02 harbors nicotinic acid phosphoribosyltransferases, enzymes crucial for NAD biosynthesis, along with site-specific integrases that facilitate the recombination and integration of DNA, as well as DNA damage-inducible proteins. Ph03 encodes tail fiber assembly proteins, which are involved in the construction of the phage tail structure. In contrast, Ph04 primarily encodes cold shock proteins, which regulate gene expression and negatively influence DNA template transcription termination. Additionally, Ph04 encodes γ-family DNA polymerase, a nucleotide transfer enzyme involved in DNA repair, and Clp protease, which plays a role in protein hydrolysis. The genes encoded by Ph05 mainly include the type-III secreted effector GogB, TerL proteins, DNA damage-inducible protein I, and ATP-binding proteins. Meanwhile, Ph06 and Ph07, located on plasmids, encode ATP-dependent Clp proteases and MtfA peptidases, both involved in protein hydrolysis. Additionally, DNA adenine methyltransferase activates site-specific DNA-methyltransferase (adenine-specific) activity. Prophage endopeptidases contribute to cytolysis and the viral release of the host cell. Lastly, 5-Methylcytosine-specific restriction endonuclease A is involved in activating nucleic acid endonuclease activity and nucleic acid binding (Fig S2).

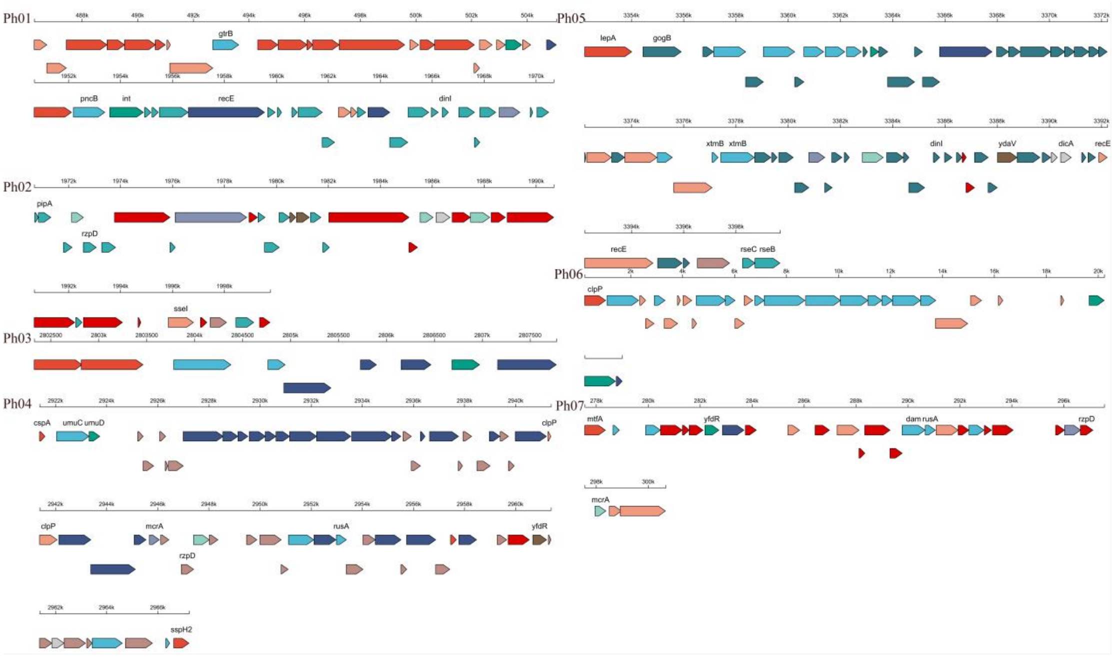

## 3 Supplementary Notes

**Supplementary Figure 3 Note:**

Note: The outermost circle represents the genome size. The second and third circles display the coding sequences (CDS) on the positive and negative strands, respectively, with different colors indicating the functional classification of the CDSs based on COGs. The fourth circle shows the positions of rRNAs and tRNAs. The fifth circle represents the GC content, where the outward red portion indicates regions with GC content higher than the average for the entire genome, with higher peaks reflecting greater deviation from the average GC content. Conversely, the inward blue portion indicates regions with GC content lower than the genome's average. The blue inward part has higher peaks corresponding to greater differences from the average GC content. The innermost circle represents the GC-Skew value, calculated as G-C/G+C. This value helps identify the leading and lagging strands of the genome, where the GC skew of the leading strand is >0 and that of the lagging strand is <0. The GC-Skew can also assist in determining the origin (cumulative offset minimum) and the termination (cumulative offset maximum) of replication. To summarize, the first and fourth circles represent CDSs on the positive and negative strands, while the second and third circles show CDSs, tRNA, and rRNA on the respective strands. The fifth circle visualizes GC content variation, and the sixth circle illustrates the GC-Skew value, which is essential for understanding replication dynamics in circular genomes. The innermost circle serves as a genome size marker.

**Supplementary Figure S1 Note:**

Note: Genomic island (gene iland, GI) represents a crucial form of horizontal transfer elements. These genomic regions harbor genes related to a variety of biological functions and are generally categorized according to their genetic content into virulence islands, drug resistance islands, metabolic islands, symbiosis islands, etc. Genomic islands are usually large, ranging in size from 10 to 200kb. They are characterized by the presence of 16-20bp forward repeats (DRs) flanking both ends of the islands and often contain functional genes, integrases, plasmid recombination-related factors, and so on. The genes carried by genomic islands frequently confer a selective advantage to the host bacteria.

**Supplementary Figure S2 Note:**

Note: Pre-phage linear mapping is depicted with arrows representing individual genes. The length of each arrow represents the length of the gene, while the direction indicates whether the gene is encoded on the sense or antisense strand. The colour of the arrows represents the COG functional classification.
